# Illness perceptions in adolescents with a psychiatric diagnosis in Pakistan

**DOI:** 10.1192/pb.bp.114.048298

**Published:** 2015-08

**Authors:** Nazish Imran, Muhammad Waqar Azeem, Mansoor R. Chaudhry, Zeeshan Butt

**Affiliations:** 1 King Edward Medical University/Mayo Hospital, Lahore, Pakistan; 2 Yale University School of Medicine, New Haven, USA; 3 Central Park Medical College, Lahore, Pakistan; 4 Prince George's Hospital Center, Cheverly, USA

## Abstract

**Aims and method** To assess adolescents' perceptions of their psychiatric illness and the role of various demographic factors in a Pakistani setting. Adolescents with various psychiatric diagnoses were interviewed using a structured questionnaire including the Illness Perceptions Questionnaire–Revised (IPQ-R).

**Results** Fifty-two adolescents with various psychiatric illnesses were interviewed; their mean age was 12.7 years and the majority (67%) were female. Males had significantly higher scores on timeline and emotional representation (*P*<0.05), suggesting strongly held beliefs about chronicity of their illness and anger and worry about their condition. Adolescents' own emotional state, stress, family problems and bad luck were endorsed by participants as some of the causal factors in their mental illness.

**Clinical implications** Despite the importance of early intervention in psychiatric problems, engaging youth in the treatment process in Pakistan remains difficult. Better understanding of how adolescents perceive their psychiatric difficulties may play a significant role in developing culturally sensitive interventions and better utilisation of services.

The prevalence of child psychiatric disorders is reported to be high in Pakistan,^1^ but engaging children and families in treatment remains challenging. Illness perceptions which are unique to different cultural groups have been found to be associated with families' engagement and help-seeking behaviours. Previous research has focused mainly on parental beliefs and identified various barriers which hinder help-seeking,^2,3^ but much remains to be known about how adolescents themselves perceive their illness. With changes evident in Pakistani society in the past few decades, adolescents' own views along with their families' perceptions are likely to influence their healthcare decisions. The current knowledge about Pakistani adolescents' perceptions of their illness, stigma and help-seeking behaviour is limited, despite assertions about adolescents' capability of ‘making decisions and choices on the basis of their own representations of health threats and illness’.^4^ To address this knowledge gap, the current study focuses on the beliefs of adolescents regarding their psychiatric illness and treatment in the context of Pakistan's unique cultural, religious and social context and attempts to determine whether various demographic factors have any role in these perceptions. Insight into these factors may help in addressing the underutilisation of services by young people in Pakistan and to identify ways of engaging them in effective treatments.

## Method

The study was approved by the Institutional Review Board of King Edward Medical University (KEMU). Participants were 11- to 18-year-old adolescents recruited from Child and Family Psychiatry Department of the KEMU/Mayo Hospital out-patient services. The adolescents included in the study had axis I psychiatric diagnoses (conversion disorder, major depression, schizophrenia, bipolar affective disorder, obsessive–compulsive disorder, generalised anxiety disorder and conduct disorder) and were taking psychotropic medications. Adolescents who were acutely ill or had intellectual disability, attention-deficit hyperactivity disorder (ADHD) and pervasive developmental disorders were excluded from the study because of the developmental nature of these disorders. Following informed consent from parents and assent from adolescents, a questionnaire was used to collect the demographic and relevant clinical information. The ICD-10 diagnostic criteria were used to assess the psychiatric diagnosis.^5^ The Illness Perceptions Questionnaire-Revised (IPQ-R) was used to glean the young people's perceptions of their illness.^6^ IPQ-R has various subscales reflecting different dimensions of illness perceptions: timeline (chronicity of illness), consequences (impact of illness on adolescent life), personal control (perception of their own control of their illness), treatment control (controllability of illness by treatment), illness coherence (adolescents' own understanding of their illness), timeline cyclical (cyclical nature of illness), emotional representations (feeling of emotions such as sadness, anger about their illness) and causes. Each statement is rated on a 5-point Likert scale ranging from 1, strongly disagree to 5, strongly agree, and addition of scores of various items yields subscale scores, except for causes in which individual items are scored as such. IPQ-R has been used in previous studies of adolescents with mental illness.^7^ The Cronbach alpha ranged from 0.79 for the timeline cyclical dimension to 0.89 for the timeline acute/chronic dimension. The questionnaire was administered to adolescents in an interview format to eliminate literacy concerns. Data were analysed using SPSS version 20. Descriptive statistics were used for socio-demographic information, and means and standard deviations of different subscales of IPQ-R were calculated. Independent samples *t*-test was used to compare the means of IPQ-R subscales across categories of binary variables such as gender, address etc.; *P*<0.05 was considered as significant.

## Results

Fifty-two youths were interviewed, with a mean age of 12.7 (s.d. 2.13) and the majority (67%, *n* = 35) being female. More than two-thirds of participants (71%, *n* = 37) lived in urban areas and almost half of the families had a nuclear family set-up (only parents and siblings) (52%) with monthly income of less than 15 000 Pakistani rupees. Family history of psychiatric illness was present in 21% (*n* = 11) and 15.4% (*n* = 8) had a history of psychiatric admission. Mean duration of illness was 14.4 months, ranging from less than 1 month to more than 30 months. Almost half of the participants had education up until the seventh grade.

Conversion disorder with comorbid emotional difficulties (depression and anxiety severe enough to warrant the use of psychotropic medication in addition to psychological treatment) was the diagnosis in 56% (*n* = 29) of patients. Other diagnoses were major depression (17.3%, *n* = 9), schizophrenia (5.8%, *n* = 3), bipolar affective disorder (3.8%, *n* = 2), obsessive-compulsive disorder (5.8%, *n* = 3), generalised anxiety disorder (1.9%, *n* = 1) and conduct disorder (1.9%, *n* = 1).

The mean scores of illness perceptions subscales along with standard deviation are presented in Table 1. Overall, the study sample perceived their illness' nature to be chronic and cyclical. The perception of significant negative consequences as a result of their mental health difficulties and a negative emotional response were also observed. There was a general perception of having some personal control of their illness as well as a positive belief in treatment role. Adolescents generally felt that they had a coherent model/understanding of their illness.

**Table 1:** Participants' mean scores, standard deviation, median and range of subscales of IPQ-R

Cognitive processes	Items, *n* (maximum possible score)	Mean (s.d.)	Median (range)
Illness perceptions			
Timeline	6 (30)	16.4 (2.2)	16 (10)
Consequences	6 (30)	17.5 (2.4)	18 (12)
Timeline cyclical	4 (20)	11.0 (2.2)	11.5 (12)
Personal control	6 (30)	18.8 (2.2)	19 (10)
Treatment control	5 (25)	12.6 (3.2)	13 (16)
Illness coherence	5 (25)	14.8 (2.2)	15 (10)
Emotional representation	6 (30)	16.2 (3.5)	16 (10)

Table 2 shows the comparison of mean scores of IPQ-R subcategories across binary variables. Males had significantly higher scores on timeline and emotional representation, which suggests strongly held beliefs about chronicity of their illness and emotional representation (anger, worry) about the condition. Patients living in a nuclear family set-up had better beliefs about controllability of illness by treatment than those living in a joint/extended family system. The rest of the comparisons were not statistically significant.

**Table 2:** Comparison of mean scores of subcategories of IPQ-R for binary variables

	Timeline (acute/chronic)	Timeline cyclical	Consequences	Personal control	Treatment control	Illness coherence	Emotional representation
Variable	Mean (s.d.)						
Gender							
Male	17.5 (2.4)*	11.0 (2.3)	17.6 (1.7)	18.9 (1.7)	12.5 (3.3)	14.8 (2.7)	17.8 (3.4)*
Female	15.8 (1.9)	11.0 (2.2)	17.4 (2.7)	18.8 (2.5)	12.7 (3.1)	14.8 (1.9)	15.4 (3.4)

Family							
Nuclear	16.4 (2.5)	11.0 (1.8)	17.5 (1.8)	18.9 (2.2)	13.8 (3.0)*	15.4 (2.2)	16.3 (3.7)
Joint	16.4 (1.9)	10.9 (2.8)	17.5 (3.0)	18.9 (2.4)	11.0 (2.6)	14.3 (2.0)	16.2 (3.5)

Address							
Urban	16.3 (2.2)	10.9 (2.4)	17.3 (2.3)	18.8 (2.0)	12.4 (3.2)	14.5 (2.2)	15.9 (3.8)
Rural	16.3 (2.4)	10.9 (2.1)	18.0 (2.8)	19.0 (3.0)	13.0 (3.3)	15.6 (2.0)	17.0 (2.9)

Family history							
Yes	16.9 (3.0)	11.4 (1.5)	17.6 (2.2)	18.0 (2.8)	13.7 (2.6)	15.0 (2.0)	17.1 (2.9)
No	16.2 (2.0)	10.8 (2.5)	17.4 (2.5)	19.0 (1.9)	12.7 (3.0)	14.8 (2.3)	16.1 (3.7)

Previous admission							
Yes	16.1 (1.8)	10.6 (2.2)	16.7 (1.7)	17.7 (2.3)	12.6 (2.5)	16.0 (1.6)	17.3 (3.4)
No	16.4 (2.3)	11.0 (2.3)	17.6 (2.5)	19.0 (2.2)	12.6 (3.3)	14.6 (2.2)	16.0 (3.5)

IPQ-R, Illness Perceptions Questionnaire-Revised.

* *P*<0.05 (calculated by applying *t*-test)

Table 3 shows the adolescents' understanding of various factors contributing towards their illness. Various psychological factors were clearly endorsed by the majority of respondents to be the cause of their emotional difficulties.

**Table 3:** Participants' responses to illness attribution (causal) items of IPQ-R

IPQ-R causal items	Participants agreeing or somewhat agreeing to factor's contribution towards their illness *n* (%)
Psychological attributions	
Stress or worry	21 (40)
My mental attitude (e.g. thinking about life negatively)	15 (29)
Family problems or worries caused my illness	20 (39)
My emotional state (e.g. feeling down, lonely anxious, empty)	24 (46)
My personality	18 (35)

Risk factors	
Hereditary (‘it runs in my family’)	7 (13)
Diet or eating habits	10 (19)
Poor medical care in my past	21 (40)
My own behaviour	19 (37)
Aging	–
Smoking	–
Alcohol	–

Immunity	
A germ or virus	9 (17)
Pollution in the environment	10 (19)
Altered immunity	6 (12)

Accident or chance	
Chance or bad luck	20 (39)
Accident or injury	15 (29)

IPQ-R, Illness Perceptions Questionnaire-Revised.

## Discussion

The present study provides insight into how adolescents in Pakistan understand their psychiatric illnesses. We observed poor mental health literacy among the adolescents in our study with regard to the nature, treatment and prognosis of their psychiatric illness. In comparison to a study of illness perceptions among Western adolescents with mood disorder,^7^ adolescents in our study believe their illness to be more chronic with serious adverse consequences. They also showed more emotional reactions (anger, sadness and worry) and appear less optimistic about the role of treatment in controlling their symptoms. Multiple factors such as cross-cultural differences, limited knowledge of available services, myths about possible causative factors and treatment for psychiatric illnesses, negative expectations of services by families, in addition to stigma, shame and reluctance to seek treatment may play a role in these beliefs as well as in underutilisation of services.^8-10^ Furthermore, children and adolescents with behavioural and emotional disorders either do not receive treatment or do not take advantage of available services in high-income countries.^2,3^ Despite various psychoeducational measures even in high-income countries, literature suggests poor understanding among adolescents about the causes, nature and treatment of psychiatric health issues.

Depression in adolescents has been associated with most stigmatising attitudes.^11^ In a study of 8- to 18-year-olds, 28% of respondents would prefer to ‘wait for depression to go away’ and 40% ‘would try to think and act like normal’. Adolescents from higher socioeconomic status, of younger age at the start of treatment and with worries about public perception are associated with self-labelling and self-stigma.^12^ These results are of concern as adolescents' own perceptions regarding their illness, stigma of treatment and concerns regarding confidentiality are considered to play an important role in professional help-seeking attitudes and behaviours.^13^ A significant proportion of children and adolescents with behavioural and emotional disorders in high-income countries either do not receive treatment or do not take advantage of available services.^14^

Our respondents' belief of treatment being not too helpful is likely to lead to reluctance to seek help or adhere to treatment. Misconceptions about psychiatric medications being addictive, need for medications to be taken for longer periods of time and slowness in learning because of medications may all contribute towards pessimistic views regarding psychiatric treatment.^13^ The majority of families perhaps prefer to seek help from informal sources such as the family, friends, religious scholars and faith healers, rather than mental health professionals, because of stigma. As a result, treatment needs of young people with a psychiatric illness remain largely unmet. There is a great need for evolving strategies to improve adolescents' perceptions about the effectiveness of treatment and to seek professional help for their emotional problems.

Gender differences were also observed in the study sample, with males being more concerned about chronicity of their illness. They also scored high on emotional representation, suggesting that they felt more shame, anger and became upset while thinking about their emotional and behavioural problems. This may be because of society's overall expectations for males to be stronger and able to manage their illness by themselves. It is important to highlight that the study sample is mainly composed of adolescents already in contact with mental health services and thus their perceptions might be different from perceptions of adolescents in the general population. The males' perception of showing ‘emotions’ as weakness in some studies^15^ may also explain stronger feelings of shame and anger among our sample who had to seek help because of the severity of their symptoms. Gender differences have been highlighted in previous research, with boys experiencing more stigma regarding mental illness, service use and treatment.^16^ Similarly, the positive perceptions of the role of treatment in helping their symptoms we observed in females is consistent with the results of previous studies.^13,17^

The majority of adolescents in the study endorsed psychological and personal factors playing a role in their illness causation (i.e. my mental attitude, my emotional state, my personality, my own behaviour, poor medical care in my past) rather than genetic and immunological causes. Cultural differences have been observed in previous research on beliefs about causation of mental illness.^18^ In a few studies, however, adolescents did endorse genetics and biological factors, stress and personal responsibility as causes of mental illness in general.^19-21^ More negative causal attributions as compared with the general public, i.e. regarding illness as being caused by their own bad behaviour, is seen in adults and children with mental illness.^22^ These beliefs about causes of mental illness in turn influence public attitudes towards the patients, with studies reporting stigmatising attitudes, beliefs of lack of willpower and personal failure to overcome the illness.^23-26^ Children were blamed for their depression and ADHD by one in four peers in a study of stigma of mental illness among children.^11^ Factors which are beyond patient control such as genetics and other biological causes are associated with less negative perceptions.^26,27^ Patients who attribute their illness to psychological factors have been shown to express more emotional reactions to their illness, as appears to be the case in the present study sample.^6^ Another interesting finding was an endorsement of bad luck by the study sample.

Pakistani culture has specific emphasis on religion and God's will, which can affect how adolescents perceive their mental illness. This is also seen in studies of Asian and Hispanic youth.^28^ Anti-stigmatising strategies for adolescents in Pakistan need to take into account these cultural variations in beliefs about causation of these illnesses in order to address stigma as well as to develop effective, culturally sensitive psychological therapies to improve adolescents' sense of well-being.

### Limitations

There were several limitations to this study. The sample size was small and was composed of people already utilising mental health services, thus results may not be generalisable to non-service-users in the general population. Furthermore, in-depth interviews in addition to a structured questionnaire are considered more helpful in understanding illness perceptions. It would also have been helpful to look at the attitudes towards professional help-seeking and determine if illness perceptions in our sample were associated with help-seeking behaviours. There was also no control group.

Despite the limitations, the study is important mainly because of its focus on adolescents' own understanding of their illness, its causation and the role of treatment. This significant area was largely ignored until now. It is difficult to develop psychoeducational programmes for the youth in Pakistan without understanding their own conceptualisation of the difficulties they are experiencing. Because of significant cultural, social and religious differences, multiple strategies need to be applied in improving mental health literacy among the youth. The stigma of psychiatric illness and treatment needs to be addressed to improve take-up of services. Counselling provision in schools and an awareness programme prepared and delivered in collaboration with paediatricians and family physicians may be more acceptable and helpful in engaging young people and families in services. Further research with a large sample, including representation from multiple sites, and in particular qualitative studies, are needed to understand and improve adolescent illness perceptions as well as attitudes towards seeking professional mental health services.
